# Compensatory pathways in tryptophan metabolism and immune regulation following IDO inhibition

**DOI:** 10.1007/s11033-026-12707-9

**Published:** 2026-09-12

**Authors:** Isabela Gontijo, Mariana Camurça, José Roberto Kfoury

**Affiliations:** Department of Surgery, School of Veterinary Medicine and Animal Science (FMVZ), University of São Paulo (USP), São Paulo, Brazil

**Keywords:** Indoleamine 2,3 dioxygenase, IDO1 inhibition, Compensatory pathway, IDO1 inhibition, Immune regulation

## Abstract

Indoleamine 2,3-dioxygenase 1 (IDO1) is an intracellular heme-dependent enzyme that catalyzes the initial oxidation of L-tryptophan into the kynurenine pathway, thereby linking tryptophan metabolism to immune regulation. IDO1 expression is mainly induced by interferon-γ during immune activation and inflammation, and its activity helps control immune responses through tryptophan depletion and the generation of immunomodulatory metabolites. Because of its central role in immunometabolism, IDO1 has been widely investigated as a therapeutic target, and several strategies have been developed to inhibit its enzymatic activity or reduce its functional contribution to immune suppression. However, increasing evidence indicates that IDO1 inhibition may not completely interrupt tryptophan catabolism or downstream immunoregulatory signaling. Instead, IDO1 blockade can be accompanied by compensatory mechanisms that preserve metabolic and immune regulatory outputs. These mechanisms may include activity or upregulation of alternative tryptophan-catabolizing enzymes such as TDO2, IDO2, and IL4I1; metabolic diversion toward serotonin and melatonin pathways; microbiota-derived indole production; and cell-type-specific metabolic rewiring. Therefore, understanding these adaptive responses is essential for interpreting the limited efficacy of IDO1-targeted approaches and for improving therapeutic strategies directed at tryptophan metabolism. This review discusses the main compensatory pathways that may emerge under IDO1 inhibition and their relevance to developing more effective immunometabolic interventions.

**Supplementary Information:**

The online version contains supplementary material available at https://doi.org/10.1007/s11033-026-12707-9.

## Introduction

Indoleamine 2,3-dioxygenase (IDO1) is a key enzyme in the kynurenine pathway of tryptophan metabolism and plays significant roles in immune regulation, neurodegeneration, and cancer progression [1]. It is a heme-containing oxidoreductase expressed intracellularly in dendritic cells (DC), macrophages, stromal cells, and cancer cells, and is mainly induced by the cytokine interferon-γ (IFN-γ) during immune activation and inflammation [2]. This occurs in acute infections, where IDO1 helps restrict pathogen growth and modulate immune responses, and in chronic inflammatory conditions and autoimmune diseases, where it acts as a regulatory mechanism to limit excessive immune activity [3]. Additionally, it plays an important function in tolerance-related settings such as pregnancy and transplantation, where it helps prevent immune rejection [4]. Overall, IDO1 induction reflects a balance between host defense and immunoregulation, often driven or enhanced by combined inflammatory signals [5].

IDO1 is also highly expressed in the tumor microenvironment (TME), where it can induce tryptophan depletion and generate immunosuppressive metabolites, suppress T cells, promote tumor angiogenesis, recruit myeloid-derived suppressor cells (MDSCs) in a Treg-dependent manner, and facilitate metastatic progression and evasion by tumor cells [6]. For this reason, IDO1 has been extensively investigated as an immunotherapy target; however, clinical and preclinical studies show that inhibiting IDO1 does not simply halt tryptophan catabolism; instead, it may trigger compensatory activation of alternative metabolic pathways [7]. These include diverting tryptophan metabolism toward the serotonin and melatonin pathways, upregulating or compensating with alternative enzymes such as TDO2 and IDO2, activating the indole pathway mediated by the gut microbiota, and engaging enzymes like IL4I1 that produce immunomodulatory metabolites. Additionally, IDO inhibition triggers cell-type–specific metabolic rewiring that affects energy metabolism and immune signaling [7, 8].

Nevertheless, these adaptive responses are not widely known. In this review, we intend to clarify the possibly compensatory pathways under IDO1 inhibition to improve therapeutic strategies that target tryptophan metabolism.

## From tryptophan catabolism to immune regulation: the role of IDO1

**Fig. 1:**
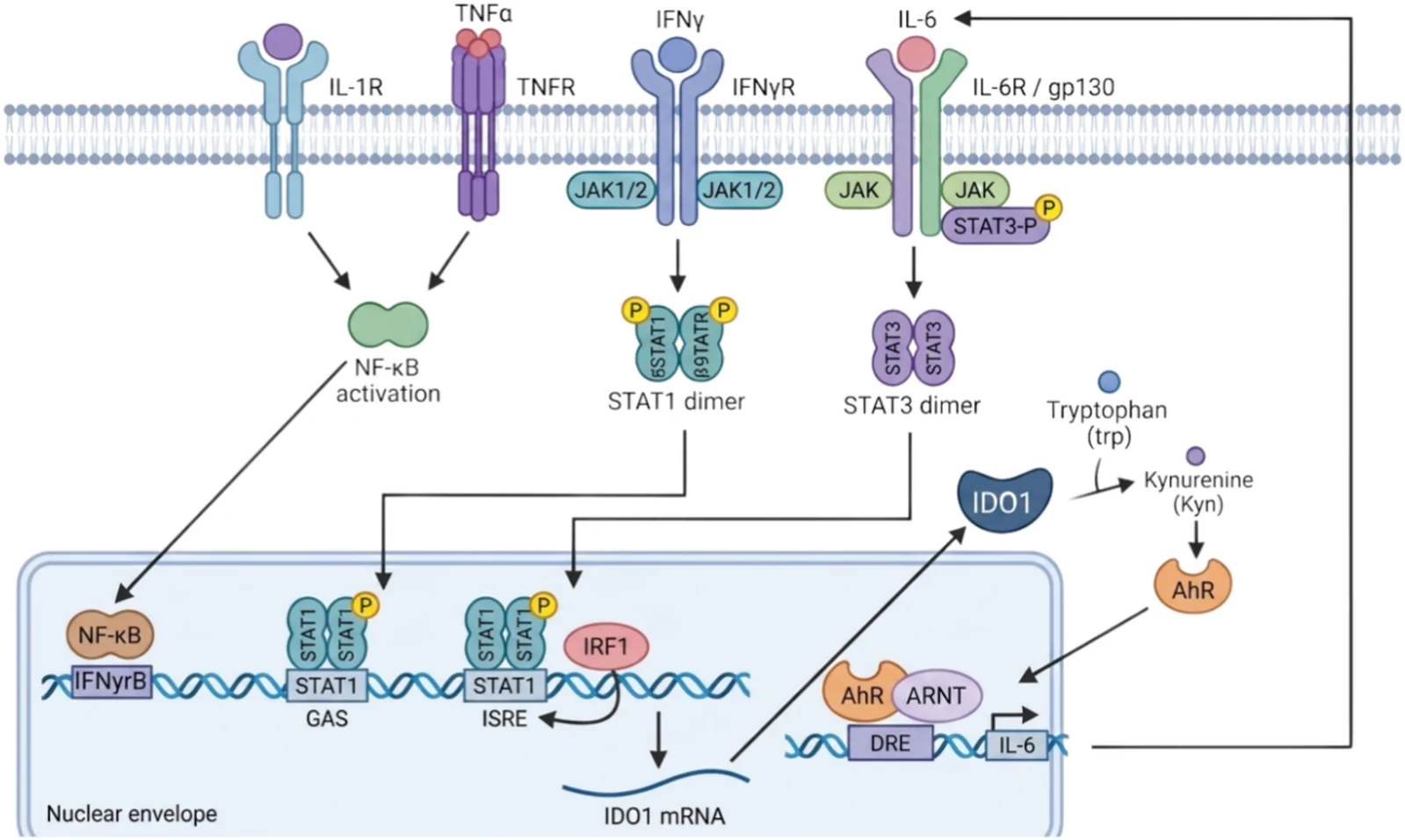
IDO1 Activation 1. Figure 1 The IDO1 Activation. AhR, aryl hydrocarbon receptor; ARNT, aryl hydrocarbon receptor nuclear translocator; DRE, dioxin response element; GAS, gamma-activated sequence; IDO1, indoleamine 2,3-dioxygenase 1; IFN-γ, interferon-gamma; IFN-γR, interferon-gamma receptor; IL-1R, interleukin-1 receptor; IL-6, interleukin-6; IL-6R, interleukin-6 receptor; IRF1, interferon regulatory factor 1; ISRE, interferon-stimulated response element; JAK, Janus kinase; JAK1/2, Janus kinase 1/2; Kyn, kynurenine; mRNA, messenger RNA; NF-κB, nuclear factor kappa B; P, phosphorylation/phosphorylated residue; STAT1, signal transducer and activator of transcription 1; STAT3, signal transducer and activator of transcription 3; STAT3-P, phosphorylated STAT3; TNF, tumor necrosis factor; TNFR, tumor necrosis factor receptor; Trp, tryptophan

IDO1 is a cytosolic, monomeric, heme-dependent enzyme that functions through two interconnected immunosuppressive axes: an enzymatic metabolic cascade and a non-enzymatic signaling cascade [9]. Its transcription is primarily induced by interferon-gamma (IFN-γ), a cytokine secreted mainly by T helper 1 (Th1) cells and natural killer (NK) cells, which activates a hierarchical signaling program leading to IDO1 gene expression [10]. This cascade begins when the IFN-γ homodimer binds to its receptor complex, composed of Interferon γ receptor type 1 (IFNGR1/alpha) and type 2 (IFNGR2/beta) chains, on the cell surface [11]. Ligand engagement induces conformational rearrangement and receptor dimerization, bringing the receptor-associated kinases JAK1 and JAK2 into proximity [12]. This enables reciprocal trans-phosphorylation and activation of JAK1/JAK2, followed by phosphorylation of specific tyrosine residues within the intracellular tail of IFNGR1 (Fig. 1) [12].

Cytosolic STAT1 monomers are recruited to the activated receptor complex through their SH2 domains, where JAK1/JAK2 catalyzes phosphorylation of STAT1 at tyrosine 701 (Tyr701). This phosphorylation acts as a molecular switch, promoting conformational changes, dissociation from the receptor, and STAT1 activation [13]. Phosphorylated STAT1 monomers then dimerize through reciprocal SH2–phosphotyrosine interactions, forming the stable STAT1 homodimer known in IFN-γ signaling as Gamma-Activated Factor (GAF) [14]. The GAF complex exposes a nuclear localization signal, allowing recognition by importin proteins, particularly importin α5 and/or importin β1, which mediate nuclear translocation through the nuclear pore complex [15].

Once inside the nucleus, GAF binds Gamma-Activated Sites (GAS), which typically follow the consensus sequence TTC(N₂–₄)GAA, within promoters of interferon-responsive genes, including.

IDO1 [16]. In the IDO1 promoter, this response is further regulated by both GAS elements and interferon-stimulated response elements (ISREs) [17]. Thus, IFN-γ induces an early STAT1/GAF-dependent transcriptional phase through GAS binding, while also promoting the expression of interferon regulatory factor 1 (IRF1), which functions as a secondary transcriptional mediator. Newly synthesized IRF1 subsequently binds ISRE motifs within the IDO1 regulatory region, amplifying and sustaining IDO1 transcriptional activation [18].

After transcriptional induction and protein expression, IDO1 becomes catalytically active only when its heme iron is maintained in the reduced ferrous state (Fe²⁺-IDO1), which is the oxygen-binding and enzymatically competent form; in contrast, the ferric form (Fe³⁺) is inactive until reduced [19]. During catalysis, molecular oxygen binds to ferrous heme, enabling IDO1 to oxidatively cleave the C2 = C3 double bond of the indole ring of L-tryptophan. This reaction generates N-formyl-L-kynurenine as the immediate product, which is then hydrolyzed by arylformamidase, also known as kynurenine formamidase or AFMID, to produce L-kynurenine [9]. This step represents the initial and rate-limiting entry point of tryptophan into the kynurenine pathway. Once formed, kynurenine can be further metabolized through downstream enzymatic branches, generating additional kynurenine-pathway metabolites and ultimately contributing to de novo (Nicotinamide Adenine Dinucleotide) NAD⁺ biosynthesis [20]. Therefore, the direct biochemical consequence of IDO1 enzymatic activity is the commitment of L-tryptophan to kynurenine-pathway catabolism, reducing the availability of free tryptophan for protein synthesis and alternative metabolic fates, including the serotonergic pathway [21].

$$\begin{aligned}\:L-Trp+{O}_{2\:}\underrightarrow{{Fe}^{2+\:}{}_{-}IDO1}\:N-formylkyrunenine\\\:\:\underrightarrow{{H}_{2}O}\:Kyrunenine\:.\:\:.\:\:.\:\:\to\:\:{NAD}^{+}/ATP\:\:\:\:\:\end{aligned}$$

As IDO1-dependent tryptophan catabolism progresses, intracellular Trp depletion is sensed as amino acid insufficiency, activating the General Control Nonderepressible 2-dependent (GCN2) integrated stress response while attenuating nutrient-sufficiency signaling through mammalian target of rapamycin complex 1 (mTORC1) [6]. Because mTORC1 integrates amino acid availability with anabolic growth, its inhibition shifts cellular metabolism away from biosynthesis, proliferation, and effector differentiation [22]. Under reduced Trp availability, this metabolic restriction limits protein translation and cell-cycle progression while promoting stress-adaptive programs, including translational reprogramming and, in some contexts, autophagy-related responses [23]. In activated T lymphocytes, diminished mTORC1 signaling impairs clonal expansion and full effector acquisition, reinforcing the antiproliferative effects of Trp starvation [24]. Thus, IDO1 activity extends beyond substrate consumption by converting inflammatory stimulation into a metabolic checkpoint in which amino acid insufficiency is sensed through both GCN2 activation and reduced mTORC1-dependent anabolic signaling, collectively favoring a restrained and more tolerogenic immune state [25].

### IDO1–AHR axis

In parallel, IDO1 enzymatic activity promotes the accumulation of L-kynurenine, which functions not only as a metabolic intermediate but also as a bioactive ligand for the aryl hydrocarbon receptor (AHR) [26]. Upon L-kynurenine binding, AHR undergoes conformational rearrangement and dissociates from its cytoplasmic chaperone complex, composed of HSP90, XAP2/AIP, and p23, thereby exposing its nuclear localization sequence and enabling nuclear translocation [27]. Once in the nucleus, AHR heterodimerizes with AHR nuclear translocator (ARNT), forming a transcriptionally active complex that binds xenobiotic response elements (XREs), also known as dioxin response elements (DREs), within regulatory regions of target genes [28]. Binding of the AHR–ARNT complex promotes the recruitment of transcriptional co-activators, general transcription factors, and RNA polymerase II, thereby initiating a distinct transcriptional program that includes canonical AHR targets such as CYP1A1, CYP1B1, and AHRR [28].

In antigen-presenting (APC) and myeloid cells, AHR-dependent transcriptional activity promotes tolerogenic reprogramming and reduces their capacity to initiate and maintain robust effector T-cell responses [29]. This attenuated antigen-presenting function limits the delivery of inflammatory, co-stimulatory, and cytokine-mediated signals required for T-cell clonal expansion, effector differentiation, cytokine secretion, and cytotoxic activity [30]. Simultaneously, AHR activation favors regulatory T-cell differentiation and/or stabilization, thereby strengthening suppressive cellular networks that counterbalance effector immunity. Within the tumor microenvironment, these effects may be further reinforced by crosstalk among Tregs, tolerogenic APCs, and suppressive myeloid populations, establishing a multicellular regulatory circuit [24].

Although AhR activation is frequently associated with immunoregulatory and tumor-promoting effects, its biological consequences depend heavily on cellular context, ligand availability, and tissue-specific transcriptional programs. In lung cancer cells, ligand-independent AhR activity has been shown to suppress invasion by promoting Smad4 degradation and reducing epithelial-to-mesenchymal transition, whereas AhR has also displayed tumor-suppressor-like properties in glioblastoma cells [31]. Therefore, modulation of the IDO1–kynurenine–AhR axis should not be interpreted as uniformly protumorigenic, as AhR activity may exert opposing functions depending on the cellular compartment and molecular context [32].

AhR can intrinsically promote the differentiation and function of resident-memory CD8⁺ T cells, including granzyme B production and polyfunctional antitumor responses [33]. AhR activity can also support NK-cell effector function, as genetic loss of AhR reduces NK-cell cytotoxic activity, whereas receptor activation can enhance IFN-γ production, cytolysis, and NK-dependent tumor control [34].

All these events result in a local immune tolerance, in which immune activation is redirected from cytotoxic effector function toward regulatory control, allowing tumor cells to persist despite the presence of inflammatory signals [24].

### The non-enzymatic IDO1 cascade

In the non-enzymatic signaling cascade of IDO1, the enzyme functions not primarily as a tryptophan-catabolizing enzyme, but as an intracellular signaling scaffold capable of sustaining long-term immune regulation [35]. This pathway is mainly associated with Transforming Growth Factor β-conditioned dendritic cells (TGF-β), particularly plasmacytoid dendritic cells, in which TGF-β signaling promotes, through a PI3K-dependent mechanism, the activation of Fyn, a Src-family tyrosine kinase responsible for phosphorylating immunoreceptor tyrosine-based inhibitory motifs located within the IDO1 sequence [36]. Phosphorylation of these Immunoreceptor Tyrosine-based Inhibitory Motif (ITIM) domains changes the functional role of IDO1 by generating docking sites for intracellular signaling molecules, especially the tyrosine phosphatases SHP-1 and SHP-2. Once recruited, SHP-1 and SHP-2 associate with phosphorylated IDO1 to form an IDO1–SHP-1/SHP-2 signaling complex, allowing IDO1 to participate directly in intracellular signal transduction independently of its catalytic activity [37].

The formation of these complexes initiates downstream regulatory signaling events that activate the non-canonical NF-κB pathway. In this pathway, the NF-κB2 precursor protein p100 is proteolytically processed into its active p52 form [38]. The generated p52 then associates with RelB, forming transcriptionally active p52/RelB complexes [39]. After their formation, these complexes translocate into the nucleus, where they bind regulatory DNA regions and promote the transcription of genes involved in sustained immune regulation [40]. These genes include IDO1 itself, TGF-β-related mediators, and type I interferon-associated factors, thereby establishing a positive regulatory feedback loop [40].

## IDO1 blockade so far

IDO1 inhibition aims to prevent the conversion of L-tryptophan into N-formyl-L-kynurenine and downstream kynurenine-pathway metabolites, thereby reducing tryptophan depletion, limiting kynurenine accumulation, preventing AHR-dependent tolerogenic transcriptional programs, and restoring effector immune activity [41]. In tumor models, blockade of the IDO–kynurenine–AHR axis has been shown to reduce kynurenine/AHR-mediated Treg differentiation and increase CD8⁺ T-cell infiltration, supporting the concept that IDO1 blockade can reverse immune tolerance within the tumor microenvironment [42].

Importantly, the biological consequences of reducing kynurenine should not be assumed to be uniformly beneficial. Although elevated kynurenine is frequently associated with immune suppression, its direct effects may differ by cell type and tissue context [43]. Kynurenine has been shown to inhibit DNA synthesis and proliferation in melanoma cells and to exert antiproliferative and pro-apoptotic effects in endothelioma cells. Moreover, in decidual NK cells, kynurenine–AhR signaling enhanced the expression of activating receptors, perforin, granzyme B, and IFN-γ, resulting in increased cytotoxic activity [44]. Although this latter observation derives from a reproductive rather than tumor context, it illustrates that kynurenine–AhR signaling is not intrinsically suppressive [44]. Consequently, therapeutic restriction of kynurenine production may relieve immunosuppressive signaling in some cellular compartments while simultaneously removing biologically protective or growth-restrictive signals in others.

The first and most direct form of IDO1 blockade is catalytic inhibition. These inhibitors bind to IDO1 and reduce its ability to oxidize L-tryptophan [45]. By blocking the catalytic step, they reduce kynurenine generation and preserve extracellular tryptophan availability [46]. This mechanism is intended to prevent activation of nutrient-stress pathways such as GCN2, restore mTORC1-dependent anabolic signaling in responding lymphocytes, and reduce kynurenine-dependent AHR activation [46]. Clinically investigated selective IDO1 inhibitors include epacadostat, navoximod, PF-06840003, LY3381916, and linrodostat/BMS-986,205; linrodostat has been described as an irreversible IDO1 inhibitor, whereas epacadostat is generally classified as a potent selective IDO1 inhibitor [47–51].

A mechanistically distinct subgroup is represented by apo-IDO1 or heme-displacing inhibitors. Instead of binding only the holoenzyme catalytic conformation, these molecules target the heme-free form of IDO1 or interfere with heme incorporation [52]. Because IDO1 requires its heme cofactor to catalyze tryptophan oxidation, displacement or prevention of heme binding can reduce enzymatic competence [48]. Recent work identified VS-15 as a compound that selectively binds the heme-free form of IDO1, inhibits enzymatic activity, and reduces IDO1-mediated signaling, indicating that apo-IDO1 targeting may interfere with both metabolic and non-enzymatic functions of IDO1 [9]. Another type of blockade is pathway modulation by tryptophan mimetics, such as indoximod, also known as D-1-methyl-tryptophan [53]. Indoximod is often discussed in the IDO1-inhibitor field, but mechanistically it is better described as an IDO pathway modulator rather than a potent direct enzymatic IDO1 inhibitor [54]. The D-isomer has been reported to act primarily as a tryptophan mimetic, reversing mTORC1 inhibition induced by tryptophan depletion in effector T cells [54]. Thus, indoximod does not simply block the catalytic site of IDO1; instead, it functionally counteracts downstream immunometabolism consequences of tryptophan starvation [55].

A broader strategy is dual or pan-pathway inhibition, in which compounds are designed to suppress more than one tryptophan-catabolizing enzyme, particularly IDO1 and TDO2 [56]. This approach is based on the rationale that tumors may compensate for IDO1 blockade by maintaining kynurenine production through TDO2 or related metabolic routes [56]. Dual IDO/TDO inhibitors such as HTI-1090, LPM-3,480,226, and M4112 have been described in clinical-development summaries. Mechanistically, this strategy aims to reduce redundancy within the kynurenine pathway by limiting kynurenine production from multiple enzymatic sources rather than targeting IDO1 alone [57–60].

Experimental approaches such as siRNA, shRNA, or CRISPR/Cas9-mediated knockout reduce IDO1 mRNA and/or protein abundance, thereby suppressing IDO1-dependent kynurenine production and attenuating downstream kynurenine-mediated signaling [61]. Because IDO1 can also act as a protein-dependent signaling molecule in selected cellular contexts, particularly through non-enzymatic scaffold functions described in tolerogenic dendritic cells, genetic reduction or deletion of IDO1 may provide broader pathway suppression than catalytic inhibition alone [41]. IDO1-targeting PROTAC strategies have also been developed to induce IDO1 protein degradation. In glioblastoma models, Bollu et al. (2022) reported NU223612, a BMS-986,205/linrodostat-derived IDO1 PROTAC, which degraded IDO1 protein in human GBM cells, inhibited IDO1 enzymatic activity and IDO1-associated NF-κB phosphorylation, and reduced IDO1 protein levels in intracranial tumors in vivo [62].

Finally, IDO1 can be targeted through immunological blockade, particularly by peptide vaccination directed against IDO1-expressing cells. This approach does not chemically inhibit the enzyme. Instead, it induces IDO1-specific T-cell responses that recognize and eliminate IDO1-expressing immunosuppressive cells [63].

These strategies differ in depth of blockade. Catalytic inhibitors primarily reduce tryptophan oxidation and kynurenine production; pathway modulators counteract downstream metabolic stress; dual inhibitors reduce compensatory kynurenine production; gene-silencing and degrader strategies reduce IDO1 abundance; and vaccination approaches eliminate IDO1-expressing suppressive cells [64]. However, the limited success of several IDO1-targeted strategies may reflect the activation of compensatory metabolic and immunoregulatory pathways that preserve kynurenine-pathway activity and maintain immune suppression even when IDO1 [65]. This possibility will be further explored in this present review, with particular emphasis on the mechanisms that may limit the effectiveness of IDO1-directed therapeutic strategies.

## Compensatory pathways activated following IDO1 blockade

### IDO2 and TDO2

Indoleamine 2,3-dioxygenase 2 (IDO2) is a paralog of IDO1 that originated from gene duplication and is encoded by a gene located adjacent to IDO1 [66]. Despite sharing structural similarity with IDO1, IDO2 displays distinct tissue distribution, catalytic properties, and immunological functions. IDO2 exhibits very low catalytic efficiency toward L-tryptophan under physiological conditions [66]. Reported kinetic analyses indicate that IDO2 has a much lower affinity for tryptophan than IDO and the expression and induction are highly context dependent. Several inflammatory stimuli have been reported to modulate IDO2 expression, but generally with weaker and more variable effects than those observed for IDO1 [67]. IFN-γ can induce IDO2 mRNA in some human mesenchymal stromal cells and certain cancer cells, whereas in human dendritic cells, IFN-γ appears to be ineffective at inducing IDO2. Other inflammatory or immunoregulatory signals, including LPS, prostaglandin E2, and IL-10, have also been reported to influence IDO2 expression or activation in selected contexts [68].

IDO2, as a heme-associated dioxygenase, can catalyze the oxidative cleavage of L-tryptophan to generate N-formyl-L-kynurenine, which is subsequently hydrolyzed by arylformamidase to produce L-kynurenine [69]. However, because IDO2 has very weak tryptophan-catabolizing activity compared with IDO1, this enzymatic route is generally considered less efficient and may not be the dominant mechanism of IDO2 function in many tissues [70]. For IDO2, the precise signaling partners and downstream pathway remain incompletely defined, but membrane localization and tyrosine phosphorylation support the possibility that IDO2 may act as a signaling-associated protein rather than only as a cytosolic metabolic enzyme [66].

Tryptophan 2,3-dioxygenase (TDO2) is a heme-dependent dioxygenase that catalyzes the first and rate-limiting step of the kynurenine pathway by oxidizing L-tryptophan into N-formyl-L-kynurenine [71]. Unlike IDO1, which is classically induced by inflammatory cytokines such as IFN-γ, TDO2 is primarily associated with constitutive and hormonally regulated tryptophan homeostasis, especially in the liver, where it contributes to systemic control of circulating tryptophan levels [72]. Structurally, human TDO2 is a heme enzyme that binds L-tryptophan and molecular oxygen at the catalytic site; crystallographic work has defined the binding modes of L-tryptophan, oxygen, and N-formylkynurenine in human TDO2 [73].

TDO2 is more strongly linked to endocrine, nutritional, and tissue-specific regulatory signals. In hepatocytes, TDO2 expression and protein stability are classically induced by glucocorticoids, and human TDO2 regulatory regions contain glucocorticoid-responsive elements that can mediate transcriptional responses to glucocorticoid signaling [72]. Substrate availability also contributes to TDO2 regulation, because tryptophan can stabilize the TDO2 protein and thereby reinforce enzymatic activity when tryptophan levels are elevated. Therefore, TDO2 functions as a hepatic sensor–effector enzyme that links dietary or systemic tryptophan abundance and glucocorticoid tone to kynurenine-pathway flux [73].

The enzymatic cascade of TDO2 begins when the catalytically competent heme center binds L-tryptophan and molecular oxygen [74]. TDO2 then catalyzes oxidative cleavage of the indole ring of L-tryptophan, generating N-formyl-L-kynurenine as the immediate product [74]. This intermediate is subsequently hydrolyzed by arylformamidase, also known as kynurenine formamidase or AFMID, to produce L-kynurenine [75]. Once generated, L-kynurenine can enter downstream kynurenine-pathway branches, producing metabolites such as kynurenic acid, 3-hydroxykynurenine, 3-hydroxyanthranilic acid, quinolinic acid, and ultimately contributing to de novo NAD⁺ biosynthesis [76, 77].

Selective inhibition of IDO1 therefore reduces one major enzymatic entry route into the kynurenine pathway but does not eliminate the substrate itself or prevent other tryptophan dioxygenases from catalyzing the same initial reaction [62]. This enzymatic redundancy provides the biochemical basis for compensation by TDO2.

When IDO1 catalytic activity is inhibited, the fraction of L-tryptophan that would normally be consumed by IDO1 remains available to other enzymes. If TDO2 is expressed and catalytically active, it can bind this available L-tryptophan together with molecular oxygen at its heme-containing catalytic center and oxidize the indole ring, producing N-formyl-L-kynurenine [42, 45]. This intermediate is subsequently hydrolyzed by arylformamidase to generate L-kynurenine, thereby reconnecting tryptophan metabolism to the same downstream kynurenine pathway described for IDO1 [46]. Thus, TDO2 does not require restoration of IDO1 activity to compensate for its inhibition; instead, it provides an alternative enzymatic entry point into the same metabolic cascade.

Inhibition of IDO1 may reduce the magnitude of IDO1-driven tryptophan depletion and consequently attenuate amino-acid stress signaling through GCN2 and mTORC1. However, if TDO2 continues to convert L-tryptophan into kynurenine, reduction of the substrate-depletion response does not necessarily imply equivalent suppression of kynurenine-dependent signaling. Residual kynurenine can remain available as an endogenous ligand for AhR, allowing activation of the AhR–ARNT transcriptional pathway even when IDO1 itself is pharmacologically inhibited [74]. Therefore, the compensatory effect of TDO2 can be understood as preservation of downstream metabolic signaling through enzymatic substitution at the initial tryptophan-oxidation step.

### Rerouting of tryptophan metabolism

A distinct form of metabolic compensation following IDO1 inhibition arises from redistribution of the available tryptophan pool toward pathways that do not generate kynurenine. When IDO1-dependent consumption decreases, a larger fraction of intracellular and extracellular L-tryptophan remains available to competing enzymes [7]. The metabolic consequence is therefore determined not only by the loss of IDO1 activity, but also by the expression and catalytic capacity of alternative tryptophan-utilizing enzymes. In cells expressing tryptophan hydroxylase (TPH), increased substrate availability can favor entry of L-tryptophan into the serotonergic pathway, thereby shifting metabolic flux away from kynurenine production [78].

The first and rate-limiting step of this pathway is catalyzed by TPH1 or TPH2, which hydroxylate L-tryptophan at the C5 position of the indole ring to generate 5-hydroxytryptophan (5-HTP) [79]. This reaction is catalyzed by a non-heme iron-dependent monooxygenase and requires molecular oxygen and tetrahydrobiopterin (BH4) as cofactors. 5-HTP is subsequently decarboxylated by aromatic L-amino acid decarboxylase (AADC) to generate serotonin (5-hydroxytryptamine; 5-HT) [79]. Thus, metabolic rerouting after IDO1 inhibition does not reproduce the original kynurenine pathway reaction; instead, it redirects the same substrate into a chemically and functionally distinct metabolite pool.

In this scenario, serotonin itself functions as an extracellular signaling molecule. Immune cells, including monocytes/macrophages, dendritic cells, and lymphocyte subsets, express different combinations of 5-HT receptor subtypes [80]. Most of these receptors are G-protein-coupled receptors, whereas 5-HT3 functions as a ligand-gated ion channel. Consequently, changes in local serotonin availability can alter intracellular signaling, cytokine production, cellular activation, migration, and differentiation in a receptor- and cell-type-dependent manner [81]. Therefore, reduction of kynurenine production following IDO1 inhibition does not necessarily eliminate tryptophan-dependent immune signaling; rather, the signaling output can shift from kynurenine/AhR-dependent regulation toward serotonergic receptor-mediated responses [81].

Serotonin can also serve as a metabolic precursor for additional bioactive compounds. In cells expressing the required biosynthetic enzymes, serotonin is acetylated by arylalkylamine N-acetyltransferase (AANAT) to form N-acetylserotonin, which is subsequently methylated by acetylserotonin O-methyltransferase (ASMT) to generate melatonin [82]. Because melatonin can modulate inflammatory signaling, cytokine production, oxidative stress, and immune-cell function, this downstream branch provides an additional mechanism through which redistribution of tryptophan metabolism may alter immune regulation after IDO1 blockade [82]. Importantly, however, engagement of the serotonin–melatonin pathway is tissue- and cell-dependent and requires expression of the corresponding enzymatic machinery.

Pharmacological IDO1 inhibition has been associated with reduced kynurenine-pathway activity accompanied by increased utilization of tryptophan through the serotonin pathway [7]. These observations indicate that inhibition of a dominant tryptophan-consuming route can modify competition for the substrate and reorganize downstream metabolite production rather than simply terminating tryptophan metabolism. In this context, metabolic compensation reflects a change in pathway identity: the loss of IDO1-dependent kynurenine production alters the relative accessibility of L-tryptophan to alternative enzymes, generating a new set of bioactive metabolites with distinct downstream signaling properties.

### ILA4I1

Beyond the alternative dioxygenases that preserve kynurenine production, interleukin-4-induced-1 (IL4I1) provides a mechanistically distinct route through which tryptophan-dependent immunoregulatory signaling can be maintained [83]. IL4I1 is a secreted flavin adenine dinucleotide (FAD)-dependent L-amino acid oxidase that catalyzes the oxidative deamination of aromatic amino acids, including L-tryptophan. In this reaction, L-tryptophan is converted into indole-3-pyruvic acid (I3P), with the concomitant production of ammonia and hydrogen peroxide [83]. Because this reaction is biochemically distinct from the heme-dependent dioxygenation catalyzed by IDO1 and TDO2, selective inhibition of IDO1 does not directly prevent IL4I1-mediated tryptophan oxidation.

IL4I1-dependent tryptophan metabolism generates I3P and related indole derivatives, including indole-3-aldehyde (I3A), that can function as endogenous AhR ligands [84]. Experimental evidence from inflammatory models has shown that cytokine-induced IL4I1 expression increases I3P and I3A production and that these metabolites activate AhR-dependent transcription [83]. Thus, IL4I1 bypasses the IDO1-dependent kynurenine pathway at the metabolic level while converging on the same downstream receptor.

Reduction of IDO1-derived kynurenine would be expected to decrease one source of endogenous AhR ligands. However, it does not eliminate AhR itself or prevent ligand generation by metabolically independent enzymes. Because IL4I1 remains catalytically independent of selective IDO1 inhibition, IL4I1-derived indole metabolites can maintain AhR-responsive signaling even when the canonical IDO1–kynurenine input is reduced [85]. Recent experimental studies outside the cancer setting further demonstrate the functional relevance of this axis: IL4I1-derived I3P and I3A activated AhR-dependent TSG-6 expression in cytokine-primed human muscle stem cells, while IL4I1/AhR signaling has also been experimentally linked to macrophage polarization and inflammatory regulation [85].

### Cell-type-specific metabolic rewiring

Cell-type-specific metabolic rewiring represents a distinct form of compensation in which inhibition of IDO1 in one cellular compartment modifies, rather than uniformly suppresses, tryptophan metabolism across the surrounding tissue [86]. The mechanistic basis for this response is the heterogeneous distribution of tryptophan transporters and metabolizing enzymes among different cell populations. Because extracellular L-tryptophan constitutes a shared metabolic pool, reduced consumption by IDO1-expressing cells increases the fraction of substrate that remains accessible to neighboring cells. The resulting redistribution of substrate can therefore shift metabolic activity toward cellular populations that retain a greater capacity for tryptophan uptake and utilization [86].

Access to this shared substrate is controlled in part at the plasma membrane. L-tryptophan must be transported into cells before entering intracellular metabolic pathways, and the abundance and activity of amino-acid transporters differ considerably among cell types and activation states. Experimental studies have shown that inflammatory or metabolic stimulation can increase tryptophan transport together with the expression of enzymes involved in its catabolism [87]. Consequently, when IDO1-dependent consumption is reduced in one cellular population, neighboring cells with high transporter activity can function as alternative metabolic sinks, capturing the newly available tryptophan and redirecting it according to their own enzymatic repertoire [87]. Compensation therefore occurs not because every cell activates the same alternative pathway, but because the relative contribution of different cellular compartments to total tissue tryptophan metabolism changes.

This redistribution can also extend from substrate consumption to metabolite exchange. Tryptophan-derived metabolites generated by one cell population can enter the extracellular environment and subsequently be taken up by other cells, thereby separating metabolite production from its biological response. For example, tryptophan availability regulates expression of the System L transporter SLC7A5/LAT1 in activated T cells, and changes in transporter abundance modify cellular uptake of kynurenine and the magnitude of downstream signaling [24]. Thus, a cell that does not itself maintain substantial tryptophan-catabolic activity can nevertheless respond to metabolites produced by neighboring cells. In this model, metabolic compensation becomes non-cell-autonomous: substrate consumption, metabolite production, and metabolite sensing may occur in different cellular compartments [24].

Recent single-cell analyses support this compartmentalized organization of tryptophan metabolism. In inflammatory intestinal tissue, tryptophan-metabolism-associated activity was distributed unevenly among cellular populations, with macrophages, fibroblasts, and epithelial cells exhibiting substantially different metabolic profiles [86]. Experimental studies in macrophages further demonstrate that changes in cellular state can induce coordinated increases in tryptophan uptake and metabolism, establishing a functional metabolic program rather than an isolated change in enzyme expression [87]. These observations indicate that the response to IDO1 inhibition should therefore be considered at the tissue-network level: suppression of IDO1-dependent flux in one population may be accompanied by altered substrate uptake, metabolite production, or metabolite sensing in another.

Accordingly, cell-type-specific metabolic rewiring may provide a mechanism of compensation following IDO1 inhibition without requiring restoration of IDO1 activity itself. The compensatory unit can therefore extend beyond an individual cell to the multicellular metabolic network, as tryptophan metabolism is heterogeneously distributed among cellular populations within the same tissue [86]. Changes in local tryptophan availability can modify transporter-dependent substrate and metabolite uptake, while the metabolic fate of the imported substrate is determined by the enzymatic program of the receiving cell [24, 87]. Consequently, inhibition of a single enzymatic source may alter the cellular distribution of tryptophan metabolism without necessarily eliminating all downstream metabolic and immunoregulatory outputs.

### Microbiota-derived indole pathways

Microbiota-derived tryptophan metabolism may contribute to the persistence of tryptophan-dependent immunoregulatory signaling after IDO1 inhibition by generating bioactive indole metabolites independently of the host IDO1–kynurenine pathway [88]. Pharmacological or genetic suppression of IDO1 reduces this host-derived kynurenine flux. However, IDO1 inhibition does not remove L-tryptophan from the tissue or intestinal environment [ 89, 90]. By reducing host IDO1-dependent kynurenine production, IDO1 inhibition may shift tryptophan metabolism away from the host kynurenine pathway and toward microbiota-associated indole production, thereby preserving tryptophan-derived AhR signaling through bacterial rather than host enzymatic routes [90, 91].

Gut microbes can use L-tryptophan as a substrate for non-canonical indole-producing pathways. Depending on the bacterial species and enzymatic repertoire, tryptophan can be converted into indole derivatives such as indole-3-aldehyde, indole-3-lactic acid, indole-3-acetic acid, indole, skatole, and indole-3-propionic acid [90]. Lactobacillus-associated taxa are particularly linked to indole-3-aldehyde and indole-3-lactic acid production, whereas other commensal anaerobes and facultative bacteria contribute additional indole metabolites [92]. These reactions occur through microbial enzymes such as tryptophanases, aromatic amino-acid aminotransferases, decarboxylases, dehydratases, and reductases, depending on the bacterial species [93]. Therefore, when IDO1-dependent kynurenine production is reduced, the metabolic “pressure” on tryptophan can be redirected toward microbial indole metabolism rather than eliminating tryptophan-derived signaling [94].

Importantly, microbial tryptophan metabolism should not be interpreted exclusively as a mechanism that restores immunosuppressive signaling after IDO1 inhibition. Microbiota-derived metabolites can also enhance antitumor immunity. Jia et al. demonstrated that metabolic cooperation between *Lactobacillus johnsonii* and *Clostridium sporogenes* increased production of indole-3-propionic acid (IPA), which enhanced the efficacy of anti-PD-1 therapy across melanoma, breast cancer, and colorectal cancer models [95]. Mechanistically, IPA increased H3K27 acetylation at the *Tcf7* super-enhancer and promoted the maintenance of progenitor exhausted CD8⁺ T cells, thereby preserving a T-cell population capable of responding to immune-checkpoint blockade [95].

Microbiota-derived indole compounds can also activate AhR in epithelial cells, innate lymphoid cells, macrophages, dendritic cells, and T-cell subsets. Zelante et al. (2013) showed that microbiota-derived tryptophan catabolites, particularly indole-3-aldehyde produced by lactobacilli, engage AhR and promote IL-22-dependent mucosal immune regulation; importantly, their study used Ido1⁻/⁻ mice and dietary tryptophan feeding to examine links among host tryptophan catabolism, microbiota, AhR, and IL-22 signaling. In this model, IDO1 inhibition decreases the host kynurenine arm, but microbial indole metabolism may preserve the downstream receptor-level output. After microbial indoles are produced in the intestinal lumen, they can act locally on epithelial or mucosal immune cells, or enter systemic circulation and influence distant tissues [90]. Lamas et al. (2020) showed that impaired microbial conversion of tryptophan into AhR ligands is associated with intestinal inflammation, whereas a tryptophan-enriched diet, AhR-ligand-producing *Lactobacillus reuteri*, or pharmacological AhR activation reduced gluten-driven immunopathology in mice.

Recent original studies also support the idea that substrate availability shapes microbial production of tryptophan-derived AhR ligands. Koper et al. (2022) used an in vitro simulator of the human intestinal microbial ecosystem and showed that tryptophan supplementation increased several microbial tryptophan metabolites and altered microbial composition, including increased relative abundance of lactobacilli in the transverse colon compartment. High residual tryptophan itself reduced AhR reporter activity, indicating that the final AhR output depends not simply on metabolite abundance, but on the balance between tryptophan, microbial agonists, and possible antagonistic effects of excess substrate [96].

Renga et al. (2023) showed that the microbiota and metabolites shape immune and metabolic activity in fungal pneumonia and reported that fecal microbiota transfer increased AhR activity in the lung. They also noted that kynurenines and downstream metabolites could be detected under conditions of relative host-pathway disruption, pointing to microbial contributions to tryptophan-derived metabolite pools [95].

## Discussion

The evidence discussed throughout this review indicates that incomplete responses to IDO1 inhibition should not be interpreted exclusively as failure to suppress the catalytic activity of the target. Rather, IDO1 operates within a distributed tryptophan-metabolic network in which substrate availability, alternative enzymes, intracellular signaling, cell-specific metabolic programs, downstream receptor activation, and microbial metabolism can each preserve different components of the biological output originally associated with IDO1. Importantly, these compensatory mechanisms are mechanistically distinct. The central question is therefore no longer whether IDO1 has been inhibited, but which components of the broader network remain capable of maintaining tryptophan-dependent signaling after inhibition.

### Context-dependent predominance of compensatory pathway

A major implication of the available evidence is that there is unlikely to be a universal compensatory response to IDO1 inhibition. Instead, the dominant mechanism should depend on the pre-existing metabolic architecture of the tissue, the cellular source of IDO1, the availability of alternative enzymes, inflammatory signaling, and the treatment context. This principle is supported by recent clinical and experimental observations showing that IDO1-associated metabolism is highly context dependent. In a phase 1/2 study of linrodostat combined with nivolumab with or without ipilimumab, pharmacological treatment reduced kynurenine across patient groups regardless of therapeutic response. In contrast, response was associated with an IFN-γ transcriptional signature, and, among non-melanoma cohorts, the combination of low TDO2 expression and high IFN-γ signaling was associated with response [96]. These findings are particularly informative because they indicate that suppression of IDO1-dependent kynurenine production alone is insufficient to define the biological state of the pathway: the expression of a parallel enzyme and the inflammatory context can modify the consequences of target inhibition [96].

The cellular origin of tryptophan metabolism may be equally important. In multiple myeloma, recent experimental work showed that malignant cells with little or no intrinsic IDO1 expression can induce IDO1 in neighboring stromal cells through a JAK–STAT1–NF-κB–IRF1-dependent mechanism [18]. Conversely, in macrophages, efferocytosis induces a coordinated program involving tryptophan transport, IDO1 expression, kynurenine production, and AhR signaling that contributes to tissue resolution [87]. These observations illustrate that IDO1 activity can originate from different cellular compartments and can participate in distinct biological programs according to tissue context. Consequently, the same pharmacological intervention may have different network-level effects depending on whether tryptophan metabolism is predominantly maintained by epithelial cells, stromal cells, macrophages, other immune populations, or a combination of these compartments.

Some compensatory events can theoretically operate immediately after catalytic inhibition because they do not require new gene expression. Pre-existing TDO2 activity, redistribution of the available tryptophan pool, residual AhR ligands, or signaling by an already present IDO1 protein can continue despite rapid suppression of IDO1-dependent catalysis. Indeed, epacadostat has been experimentally shown to inhibit kynurenine production while enhancing association of IDO1 with SHP phosphatase, and subsequent work demonstrated persistent stabilization of a signaling-competent apo-like IDO1 state after catalytic inhibition [46, 97]. In contrast, transcriptional induction of alternative enzymes, remodeling of transporter expression, changes in cellular composition, and microbiota restructuring would be expected to develop over longer periods. This suggests a possible progression from immediate biochemical redundancy to later transcriptional adaptation.

Most available studies examine individual compensatory mechanisms in different experimental systems rather than following multiple pathways longitudinally after IDO1 inhibition. It therefore remains uncertain whether TDO2 activation, metabolic rerouting, IL4I1-dependent signaling, non-enzymatic IDO1 activity, and microbial adaptation occur simultaneously, sequentially, or only in mutually exclusive biological contexts. Addressing this question will require longitudinal experimental designs combining serial metabolomics with transcriptional, proteomic, and cell-resolved analyses before and after IDO1 inhibition. Such studies would allow compensation to be distinguished from pre-existing redundancy and would establish whether adaptive pathways emerge as early pharmacodynamic events or as acquired responses to sustained metabolic pressure.

### From enzyme-specific inhibition to network-based regulation

These observations support a shift in therapeutic interpretation from inhibition of a single enzyme toward regulation of the total tryptophan-metabolic network. A useful distinction is that between target engagement and network suppression. Target engagement asks whether the inhibitor successfully reduces IDO1 catalytic activity, network suppression asks whether the biological outputs produced by the wider system are also reduced.

In a randomized phase 2 study in metastatic non-small-cell lung cancer, epacadostat reduced circulating kynurenine when combined with pembrolizumab, yet the addition of epacadostat did not improve objective response compared with pembrolizumab alone [98]. Thus, successful suppression of an IDO1-dependent metabolite can occur without equivalent therapeutic benefit. This discrepancy is consistent with the possibility that residual kynurenine production, alternative AhR ligands, non-enzymatic signaling, or other network components continue to support biological outputs after catalytic blockade.

The same concept explains why the relevant unit of therapeutic intervention may differ among biological contexts. Where TDO2 is highly expressed, suppressing IDO1 alone leaves a parallel enzymatic entry point into the kynurenine pathway. Where alternative metabolites maintain AhR activity, the critical resistant node may lie downstream of IDO1 rather than at the initial catalytic step. Where apo-IDO1 signaling persists, inhibiting the catalytic pocket leaves the protein itself available as a signaling scaffold. Conversely, in microbiota-rich interfaces, the metabolic network extends beyond host cells altogether. Host–microbiota tryptophan partitioning has been experimentally demonstrated in inflammatory disease, and microbial cross-feeding can transform one bacterial tryptophan metabolite into another, indicating that microbial compensation itself can operate as a community-level metabolic network rather than through a single bacterial pathway [87].

Protein degradation represents one possible approach to overcome the distinction between catalytic inhibition and persistence of the IDO1 protein. Recent optimization of an IDO1-targeting PROTAC generated NU227326, which induced potent and sustained IDO1 degradation in human glioblastoma cells through the ubiquitin–proteasome system [99]. Conceptually, removal of IDO1 could suppress both its catalytic and protein-dependent functions, whereas conventional catalytic inhibitors address primarily substrate oxidation. Nevertheless, degradation of IDO1 would not eliminate TDO2, IL4I1, microbial metabolism, or downstream AhR activation. Therefore, increasingly broad inhibition is not necessarily synonymous with improved therapy. The goal should instead be to identify the compensatory node that dominates in the relevant tissue and disease state while avoiding unnecessary disruption of beneficial tryptophan-dependent functions in other compartments.

### Therapeutic combination strategies and clinical monitoring of compensatory responses

When persistent kynurenine production is associated with high TDO2 expression, simultaneous targeting of IDO1 and TDO2, or targeting of a downstream shared output, may be more rational than increasing the dose of an IDO1-selective inhibitor. When catalytic inhibition leaves non-enzymatic IDO1 signaling intact, strategies that destabilize or degrade IDO1, or compounds capable of interfering with both catalytic and signaling functions, may provide greater mechanistic coverage. In contexts characterized by strong IL4I1-derived AhR activity, targeting the alternative metabolic source or the common receptor-level output could be considered. However, because AhR integrates numerous endogenous, dietary, cellular, and microbial ligands, systemic AhR inhibition may produce substantially broader biological effects and will require careful assessment of tissue specificity.

Recent clinical studies also indicate that patient selection will be essential. In resectable head and neck squamous cell carcinoma, addition of BMS-986,205 to nivolumab did not improve radiographic response across the entire cohort, but greater pathological treatment response was observed among patients with high baseline IDO1 RNA expression. Moreover, predictive transcriptional features differed according to HPV status: IFN-γ, PD-L1 and NK-cell-related signatures were associated with response in HPV-negative disease, whereas B-cell and cancer-associated fibroblast signatures distinguished response in HPV-positive tumors [100]. Together with the linrodostat study, in which low TDO2 combined with high IFN-γ signaling was associated with response, these findings suggest that expression of IDO1 alone is unlikely to be sufficient for patient stratification [98].

Monitoring compensation clinically will therefore require biomarkers at multiple levels of the network. Circulating tryptophan, kynurenine, and the kynurenine/tryptophan ratio remain useful pharmacodynamic measurements of pathway engagement and can be quantified longitudinally by mass spectrometry. However, because kynurenine reduction does not reliably predict therapeutic response, metabolite measurements should be complemented by tissue-level assessment of IDO1 and TDO2 expression and, where relevant, IL4I1 [96, 98]. Measurement of AhR-responsive transcriptional markers may help determine whether downstream signaling remains active despite reduced kynurenine. Importantly, the cellular origin of these signals should also be resolved whenever possible. Spatial transcriptomics, multiplexed tissue imaging, and single-cell approaches could distinguish enzyme expression in malignant, stromal, endothelial, and immune compartments and determine whether the source of tryptophan metabolism changes during treatment.

A rational translational design would compare pretreatment samples with early and subsequent on-treatment samples to determine whether inhibition produces the expected decrease in IDO1-dependent flux and whether alternative pathways emerge thereafter. Serial plasma metabolomics could detect changes in tryptophan, kynurenine, serotonin-related metabolites, and other relevant products, tumor or tissue biopsies could assess IDO1/TDO2/IL4I1 expression and AhR transcriptional activity, and, in settings where host–microbiota interactions are biologically plausible, stool metagenomics combined with microbial metabolomics could determine whether changes in host metabolism are accompanied by altered indole production.

Future studies should therefore prioritize longitudinal and compartment-resolved experimental designs capable of measuring several compensatory pathways simultaneously. Multi-omic profiling before and during IDO1 inhibition could establish whether a patient begins with a TDO2-dominant, IL4I1/AhR-dominant, non-enzymatic IDO1-dominant, cell-compartmentalized, or microbiota-influenced metabolic state and whether that state changes under therapeutic pressure. Adaptive clinical trial designs could subsequently use these molecular responses to select or modify combination strategies rather than applying the same combination to biologically heterogeneous populations.

Overall, the available evidence supports a model in which IDO1 inhibition perturbs a dynamic network rather than switching off an isolated metabolic pathway. The limited efficacy of selective catalytic blockade may therefore reflect both pre-existing redundancy and adaptive compensation occurring at enzymatic, receptor, cellular, protein-signaling, and microbial levels. The next generation of IDO1-directed strategies should move beyond confirmation of target inhibition toward identification of the pathway that preserves the relevant biological output after inhibition. Defining where, when, and in which cellular compartment compensation occurs will be essential for selecting rational combination partners, developing informative biomarkers, and determining which patients are most likely to benefit from therapeutic modulation of tryptophan metabolism.

## Supplementary Information

Below is the link to the electronic supplementary material.

Supplementary Material 1

## Data Availability

No datasets were generated or analysed during the current study.

## Abbreviations

5-HT: 5–Hydroxytryptamine (serotonin)
5-HT3: 5–Hydroxytryptamine receptor 3
5-HTP: 5–Hydroxytryptophan
AADC: Aromatic L–amino acid decarboxylase
AANAT: Arylalkylamine N–acetyltransferase
AFMID: Arylformamidase / kynurenine formamidase
AhR / AHR: Aryl hydrocarbon receptor
AHRR: Aryl hydrocarbon receptor repressor
AIP: AHR–interacting protein
anti-PD–1: Anti–programmed cell death protein 1 antibody/therapy
APC: Antigen–presenting cell
ARNT: Aryl hydrocarbon receptor nuclear translocator
ASMT: Acetylserotonin O–methyltransferase
BH4: Tetrahydrobiopterin
CD8⁺: Cluster of differentiation 8–positive
CRISPR/Cas9: Clustered regularly interspaced short palindromic repeats / CRISPR–associated protein 9
CYP1A1: Cytochrome P450 family 1 subfamily A member 1
CYP1B1: Cytochrome P450 family 1 subfamily B member 1
DC: Dendritic cell
DNA: Deoxyribonucleic acid
DRE: Dioxin response element
FAD: Flavin adenine dinucleotide
Fe²⁺: Ferrous iron
Fe³⁺: Ferric iron
Fyn: FYN proto–oncogene, Src family tyrosine kinase
GAF: Gamma–activated factor
GAS: Gamma–activated site
GBM: Glioblastoma
GCN2: General control nonderepressible 2
H3K27: Histone H3 lysine 27
HPV: Human papillomavirus
HSP90: Heat shock protein 90
I3A: Indole–3–aldehyde
I3P: Indole–3–pyruvic acid
IDO: Indoleamine 2,3–dioxygenase
IDO1: Indoleamine 2,3–dioxygenase 1
Ido1: Murine gene symbol for IDO1
IDO2: Indoleamine 2,3–dioxygenase 2
IFN-γ: Interferon–gamma
IFN-γR: Interferon–gamma receptor
IFNGR1: Interferon gamma receptor 1
IFNGR2: Interferon gamma receptor 2
IL-1R: Interleukin–1 receptor
IL-6: Interleukin–6
IL-6R: Interleukin–6 receptor
IL-10: Interleukin–10
IL-22: Interleukin–22
IL4I1: Interleukin–4–induced–1
IPA: Indole–3–propionic acid
IRF1: Interferon regulatory factor 1
ISRE: Interferon–stimulated response element
ITIM: Immunoreceptor tyrosine–based inhibitory motif
JAK: Janus kinase
JAK1 / JAK2: Janus kinase 1 / Janus kinase 2
Kyn: Kynurenine
LAT1: L–type amino acid transporter 1
LPS: Lipopolysaccharide
MDSCs: Myeloid–derived suppressor cells
mRNA: Messenger RNA
mTORC1: Mechanistic target of rapamycin complex 1
NAD⁺: Nicotinamide adenine dinucleotide
NF-κB: Nuclear factor kappa B
NF-κB2: Nuclear factor kappa B 2
NK: Natural killer cell
P: Phosphorylation / phosphorylated residue
p23: HSP90 co–chaperone p23
p52: NF–κB2 p52 subunit
p100: NF–κB2 precursor protein p100
PD-1: Programmed cell death protein 1
PD-L1: Programmed death–ligand 1
PI3K: Phosphoinositide 3–kinase
PROTAC: Proteolysis–targeting chimera
RelB: RelB transcription factor / NF–κB family member
RNA: Ribonucleic acid
SH2: Src homology 2 domain
SHP: 1–SH2–domain–containing phosphatase 1
SHP: 2–SH2–domain–containing phosphatase 2
shRNA: Short hairpin RNA
siRNA: Small interfering RNA
SLC7A5: Solute carrier family 7 member 5
Smad4: SMAD family member 4
STAT1: Signal transducer and activator of transcription 1
STAT3: Signal transducer and activator of transcription 3
STAT3: P–Phosphorylated STAT3
T: cell–T lymphocyte
Tcf7: Gene encoding T–cell–specific transcription factor 7
TDO: Tryptophan 2,3–dioxygenase
TDO2: Tryptophan 2,3–dioxygenase 2
TGF: β–Transforming growth factor beta
Tgfb1: Gene encoding transforming growth factor beta 1
Th1: T helper type 1 cell
TME: Tumor microenvironment
TNF: Tumor necrosis factor
TNFR: Tumor necrosis factor receptor
TPH: Tryptophan hydroxylase
TPH1 / TPH2: Tryptophan hydroxylase 1 / tryptophan hydroxylase 2
Treg / Tregs: Regulatory T cell(s)
TRIM21: Tripartite motif–containing 21
Trp: Tryptophan
TSG-6: Tumor necrosis factor–stimulated gene 6 protein
Tyr701: Tyrosine 701
USP14: Ubiquitin–specific protease 14
XAP2 / AIP: X–associated protein 2 / AHR–interacting protein
XRE: Xenobiotic response element
